# Influence of anthropometric factors on tumour biological characteristics of colorectal cancer in men and women: a cohort study

**DOI:** 10.1186/1479-5876-11-293

**Published:** 2013-11-21

**Authors:** Jenny Brändstedt, Sakarias Wangefjord, Signe Borgquist, Björn Nodin, Jakob Eberhard, Jonas Manjer, Karin Jirström

**Affiliations:** 1 Department of Clinical Sciences, Division of Pathology, Lund University, Skåne University Hospital, Lund, Sweden; 2 Department of Clinical Sciences, Division of Oncology, Lund University, Skåne University Hospital, Lund, Sweden; 3 Department of Clinical Sciences, Division of Surgery, Lund University, Skåne University Hospital, Malmö, Sweden; 4 The Malmö Diet and Cancer Study, Lund University, Malmö, Sweden

## Abstract

**Background:**

Obesity is a well established risk factor of colorectal cancer (CRC), but how body size influences risk of colorectal cancer defined by key molecular alterations remains unclear. In this study, we investigated the relationship between height, weight, body mass index (BMI), waist- and hip circumference, waist-hip ratio (WHR) and risk of CRC according to expression of beta-catenin, cyclin D1, p53 and microsatellite instability status of the tumours in men and women, respectively.

**Methods:**

Immunohistochemical expression of beta-catenin, cyclin D1, p53 and MSI-screening status was assessed in tissue microarrays with tumours from 584 cases of incident CRC in the Malmö Diet and Cancer Study. Six anthropometric factors: height, weight, BMI, waist- and hip circumference, and WHR were categorized by quartiles of baseline measurements and relative risks of CRC according to expression of beta-catenin, cyclin D1, p53 and MSI status were calculated using multivariate Cox regression models.

**Results:**

High height was associated with risk of cyclin D1 positive, and p53 negative CRC in women but not with any investigative molecular subsets of CRC in men. High weight was associated with beta-catenin positive, cyclin D1 positive, p53 negative and microsatellite stable (MSS) tumours in women, and with beta-catenin negative and p53 positive tumours in men. Increased hip circumference was associated with beta-catenin positive, p53 negative and MSS tumours in women and with beta-catenin negative, cyclin D1 positive, p53 positive and MSS tumours in men. In women, waist circumference and WHR were not associated with any molecular subsets of CRC. In men, both high WHR and high waist circumference were associated with beta-catenin positive, cyclin D1 positive and p53 positive tumours. WHR was also associated with p53 negative CRC, and waist circumference with MSS tumours. High BMI was associated with increased risk of beta-catenin positive and MSS CRC in women, and with beta-catenin positive, cyclin D1 positive and p53 positive tumours in men.

**Conclusions:**

Findings from this large prospective cohort study indicate sex-related differences in the relationship between obesity and CRC risk according to key molecular characteristics, and provide further support of an influence of lifestyle factors on different molecular pathways of colorectal carcinogenesis.

## Introduction

Colorectal cancer (CRC) is one of the most common forms of human cancer worldwide with approximately 1 million new cases detected every year [1]. Numerous epidemiological studies have examined the relationship between body weight and risk of CRC, most of which have demonstrated a positive association between a high body weight and an increased risk of CRC, particularly in men [2-4]. However, CRC is a largely heterogenous disease in terms of its biological properties and accumulating evidence suggest that aetiological factors influence the carcinogenetic process differentially according to different molecular pathways [5-8].

Colorectal carcinogenesis can be regarded as a complex process with multigene participation, mainly involving at least three distinct pathogenetic pathways: chromosomal instability (CIN), microsatellite instability (MSI) and CpG island methylator phenotype (CIMP) [9,10]. The ‘suppressor’ pathway involves loss of function of the tumour suppressor genes APC (Adenomatosis Polyposis Coli gene), DCC (Deleted in Colorectal Carcinoma gene), p53, and activation of the proto-oncogene *k-ras*. This pathway accounts for approximately 65-70% of sporadic CRC [11,12] and for cancers associated with familial adenomatous polyposis (FAP), constituting less than 1% of all CRC [13].

Beta-catenin is a membrane-associated protein with essential functions in the regulation of cellular adhesion and the major mediator of the Wnt-signaling pathway [14,15]. Inactivation of kinases in the APC-complex leads to accumulation of cytoplasmic and nuclear beta-catenin, contributing to tumour progression [16-18]. Morikawa et al. have recently shown that BMI is associated with a higher risk of beta-catenin negative-, but not beta-catenin positive colorectal cancer [19]. Cyclin D1 is activated by WNT/beta-catenin signalling after mutation of the adenomatous polyposis coli gene (APC) [20]. Cyclin D1 is an important cell-cycle regulating protein and overexpression is seen in about one third of CRC [21]. Although various studies have linked the *CCND1* G870A polymorphism to increased CRC risk, the results remain controversial [22,23].

Inactivation of the p53 pathway by p53 mutations is one of the key genetic steps in colorectal carcinogenesis and approximately 40-50% of tumours in the colon have alterations in the p53 gene [24-26]. The p53 suppressor gene is involved in numerous cellular processes, including induction of apoptosis and cell-cycle arrest, and p53 also plays an important role in cellular energy metabolism [27-29]. It has been shown that reduced nutrient or energy levels induce p53 [30], and given the important role of diet and lifestyle factors to the etiology of CRC, it can be hypothesized that life style factors are associated with p53 mutations. Very few previous studies have however addressed this question. Slattery et al. have shown a positive relationship between western style diet, but not obesity, and p53 mutations [31].

The second pathway is initiated by germline mutations in the mismatch repair (MMR) genes, e.g. *MLH1*, *MSH2*, *MSH6*, and *PMS2*, or somatic tumour MLH1 promoter methylation, leading to microsatellite instability (MSI). MSI is detected in approximately 15% of sporadic CRC, predominantly tumours located in the proximal colon, and in almost all cancers from patients with hereditary non-polyposis colon cancer (HNPCC), accounting for 3-5% of all CRC [32-34]. Previous data indicate an association between obesity, MSS and risk of CRC [35].

Taken together, while it is well documented that body size influences CRC risk, also with differences regarding sex, location, and tumour stage [36], it remains unclear how this association differs according to molecular tumour phenotype.

The aim of this study was therefore to examine sex-related differences in the relationship between anthropometric factors and beta-catenin alterations, expression of cyclin D1 and p53, and MSI screening status of incident CRC in a large population based prospective cohort study (n = 584).

## Subjects and methods

### Study group

Until end of follow-up 31 December 2008, 584 incident cases of CRC had been registered in the prospective, population-based cohort study Malmö Diet and Cancer Study (MDCS) [37]. Between 1991–1996, a total number of 28 098 individuals; 11 063 (39,4%) men and 17 035 (60,6%) women, between 44–74 years where enrolled from a background population of 74 138. All participants completed the baseline examination, which included a questionnaire, anthropometric measurements and a dietary assessment. The questionnaire covered questions on physical activity, use of tobacco and alcohol, heredity, socio-economic factors, education, occupation, previous and current disease and current medication. In addition, blood samples were collected and stored in -80°C. Follow up is performed annually by record-linkage to national registries for cancer and cause of death. Cases were identified from the Swedish Cancer Registry up until 31 December 2007, and from The Southern Swedish Regional Tumour Registry for the period of 1 January to 31 December 2008. All tumours with available slides or paraffin blocks were histopathologically re-evaluated by a senior pathologist (KJ) on haematoxylin and eosin-stained slides. Histopathological, clinical and treatment data were obtained from the clinical and/or pathology records. Information on vital status and cause of death was obtained from the Swedish Cause of Death Registry up until 31 December 2009. Patient and tumour characteristics of the cohort, including specified location of colonic tumours, have been described in detail previously [38-40]. Ethical permissions for the MDCS (Ref. 51/90), and the present study (Ref. 530/2008), were obtained from the Ethics Committee at Lund University.

### Anthropometric measurements

At baseline examination, weight, (multiples of 0.1 kg) and height (to the nearest 0.005 m) were measured by a trained nurse, and body mass index (BMI) was calculated as kg/m^2^. Waist circumference was measured at the midpoint between the lower ribs and the iliac crest, and for hip circumference the level of greatest lateral extension was used. These measurements were estimated to the nearest 0.01 m. The waist and hip circumferences of each participant were used to calculate waist-hip ratio (WHR; cm/cm) as an additional measure of fat distribution.

### Tissue microarray (TMA) construction and immunohistochemistry

Tumours with an insufficient amount of material were excluded, and a total number of 557 (89.0%) tumours were suitable for TMA construction. In brief, two 1.0 mm cores were taken from each tumour and mounted in a new recipient block using a semi-automated arraying device (TMArrayer, Pathology Devices, Westminster, MD, USA). As demonstrated previously, there was no selection bias regarding the distribution of clinicopathological characteristics between the TMA cohort and the full cohort [39].

For immunohistochemical analysis, 4 μm TMA-sections were automatically pre-treated using the PT-link system (DAKO, Glostrup, Denmark) and then stained in an Autostainer Plus (DAKO, Glostrup, Denmark). MSI screening status was evaluated as previously described [41]. Immunohistochemical stainings were evaluated as negative when all tumour cells showed loss of nuclear staining. Surrounding stromal cells and tumour infiltrating lymphocytes served as internal controls for each biopsy core. A nuclear reaction of tumour cells was assessed as a positive staining. MSI screening status was defined in accordance with previous studies [41] whereby tumour samples lacking nuclear staining of MLH1, PMS2, MSH2 or MSH6 were considered to have a positive MSI screening status. Hereafter, tumours with a positive MSI screening status are referred to as MSI and tumours with negative MSI screening status are referred to as MSS.

Immunohistochemical staining of beta-catenin was performed and evaluated as previously described [42],whereby membranous staining was denoted as 0 (present) or 1 (absent), cytoplasmic staining intensity as 0–2 and nuclear staining intensity as 0–2. In this study, the analyses were limited to nuclear expression of beta-catenin. Cyclin D1 expression was evaluated as previously described [38] and p53 positivity was defined as > = 50% tumour cells with strong nuclear staining intensity in accordance with previous studies [40]. All immunohistochemical stainings were evaluated by two independent observers (SW and KJ), who were blinded to clinical and outcome data. Scoring differences were discussed in order to reach consensus.

### Statistical methods

Distribution of established and potential risk factors for CRC was compared between CRC cases and the rest of the study cohort (Table 1). Distribution of cytoplasmic and nuclear beta-catenin expression, expression of p53 and cyclin D1, and MSI-status is also shown in Table 1. Anthropometric measurements were divided into quartiles. Separate quartiles were calculated for men and women [36]. A Cox proportional hazards analysis was used in order to compare risk of CRC between different categories of anthropometric factors according to beta-catenin over-expression, p53, and cyclin D1 expression and MSI screening status according to gender and tumour location, i.e. colon vs rectum. This yielded hazard ratios (HR) with a 95% confidence interval. Follow-up time was defined as time from baseline to diagnosis, death or end of follow-up 31 December 2009. The proportional hazards assumption was confirmed by a log, - log plot [43]. In the multivariate Cox analysis potential confounders were included, i.e. age (years), educational level (not completed elementary school/elementary school (6–8 years)/ “grundskola” (9–10 years)/“studentexamen” (10–12 years)/ one year after “studentexamen”/university degree), smoking habits (yes regularly, yes occasionally, former smoker, never smoker), and alcohol consumption (g/day) (Table 1). A case-to-case analysis examined the heterogeneity between different tumour subgroups regarding their association to anthropometrics using an unconditional logistic regression model. Chi square test was applied for assessment of the distribution of investigative factors according to baseline characteristics. All statistical analyses were conducted using SPSS version 20 and 21 (SPSS Inc., Chicago, IL, USA). Trend was calculated as linear trend over quartiles. A two-tailed p-value less than 0.05 was regarded as statistically significant.

**Table 1:** Distribution of risk factors in cases and rest of cohort

**Characteristics**	**Rest of cohort n = 27514**	**CRC cases n = 584**	***p***	**beta-catenin + n = 304 (61.0%)**	**beta- catenin – n = 194 (39.0%)**	***p***	**Cyclin D1+ n = 400 (80.3%)**	**Cyclin D1 –n = 98** ** (19.7%)**	***p***	**p53 + n = 241 (48.3%)**	**p53 – n = 258 (51.7%)**	***p***	**MSI n = 71 (14.6%)**	**MSS n = 416 (85.4%)**	***p***
**Sex**			*<0.001*			*0.863*			*0.226*			*0.722*			*0.057*
Male (%)	10783 (39.2)	280 (47.9)		145 (47.7)	91 (46.9)		185 (46.2)	52 (53.1)		112 (46.5)	124 (48.1)		26 (36.6)	203 (48.8)	
Female (%)	16731 (60.8)	304 (52.1)		159 (52.3)	103 (53.1)		215 (53.8)	46 (46.9)		129 (53.5)	134 (51.9)		45 (63.4)	213	
**Age at baseline (years)**	58.0	61.8		61.9	62.0		62.3	60.2		61.5	62.3		64.4	61.5	
Male	59.2	61.7	*<0.001*	61.5	62.1	*0.430*	61.9	60.5	*0.204*	61.2	62.0	*0.452*	64.6	61.2	*0.029*
Female	57.3	62.1	*<0.001*	62.5	61.7	*0.638*	62.7	59.7	*0.001*	61.7	62.6	*0.252*	64.3	61.7	*0.024*
**Smoking male (% )**			*0.104*			*0.749*			*0.371*			*0.199*			*0.793*
Regularly	2572 (25.5)	53 (18.9)		25 (17.2)	17 (18.7)		35 (18.9)	8 (15.4)		17 (15.2)	24 (19.2)		5 (19.2)	38 (18.5)	
Occasionally	520 (4.8)	12 (4.3)		5 (3.4)	3 (3.3)		8 (4.3)	0		5 (4.4)	3 (2.4)		1 (3.89	7 (3.4)	
Former smoker	4635 (43.0)	145 (51.7)		80 (55.2)	44 (48.4)		94 (51.4)	31 (59.6)		66 (58.9)	60 (48.0)		11 (42.3)	107 (52.2)	
Never smoker	3046 (28.3)	70 (25.0)		35 (24.1)	27 (29.7)		48 (25.9)	13 (25.0)		24 (21.4)	38 (30.4)		9 (34.6)	53 (25.9)	
**Smoking female (%)**			*0.652*			*0.592*			*0.972*			*0.009*			*0.694*
Regularly	4976 (29.8)	71 (23.4)		36 (22.6)	25 (24.3)		49 (22.8)	11 (23.9)		26 (20.2)	35 (26.1)		12 (26.7)	47 (22.1)	
Occasionally	722 (4.3)	8 (2.6)		3 (1.9)	4 (3.9)		7 (3.2)	1 (2.2)		2 (1.6)	6 (4.5)		1 (2.2)	7 (3.3)	
Former smoke	4637 (27.7)	87 (28.6)		41 (25.7)	30 (29.1)		57 (26.5)	13 (28.3)		27 (20.9)	43 (32.1)		9 (20.0)	59 (27.7)	
Never smoker	7386 (44.2)	138 (45.4)		79 (49.7)	44 (42.7)		102 (47.4)	21 (45.7)		74 (57.4)	50 (37.3)		23 (51.1)	100 (46.9)	
**Level of education male (%)**			*0.912*			*0.727*			*0.760*			*0.250*			*0.451*
Not completed	85 (0.8)	2 (0.7)		1 (0.7)	0		1 (0.5)	0		1 (0.9)	0		0	1 (0.5)	
6-8 years	4852 (45.1)	137 (48.9)		76 (52.4)	41 (45.1)		93 (50.3)	25 (48.1)		57 (50.9)	61 (48.8)		13 (50.0)	102 (49.8)	
9-10 years	2113 (19.6)	55 (19.6)		28 (19.3)	21 (23.1)		35 (18.9)	14 (26.9)		24 (21.4)	25 (20.0)		4 (15.4)	44 (21.5)	
10-12 years	1279 (11.9)	29 (10.4)		16 (11.0)	10 (11.0)		21 (11.3)	4 (7.7)		7 (6.3)	18 (14.4)		6 (23.1)	20 (9.8)	
1 year university	998 (9.3)	19 (6.7)		9 (6.2)	5 (5.5)		12 (6.5)	2 (3.8)		9 (8.0)	5 (4.0)		1 (3.8)	13 (6.3)	
University degree	1424 (13.2)	38 (13.6)		15 (10.3)	14 (15.4)		23 (12.4)	7 (13.5)		14 (12.5)	16 (12.8)		2 (7.7)	25 (12.2)	
**Level of education female (%)**			*0.009*			*0.019*			*0.796*			*0.496*			*0.316*
Not completed	126 (0.8)	3 (1.0)		1 (0.6)	2 (1.9)		3 (1.4)	0		2 (1.6)	1 (0.7)		0	3 (1.4)	
6-8 years	6419 (38.5)	150 (49.8)		88 (56.4)	48 (46.6)		111 (52.4)	22 (47.8)		73 (56.6)	61 (46.2)		25 (55.6)	105 (50.0)	
9-10 years	5086 (30.5)	75 (24.9)		27 (17.3)	35 (34.0)		52 (24.5)	12 (26.1)		26 (20.2)	38 (28.8)		13 (28.9)	50 (23.8)	
10-12 years	1161 (7.0)	22 (7.3)		15 (9.6)	3 (2.9)		13 (6.1)	5 (10.9)		9 (7.0)	9 (6.8)		0	18 (8.6)	
1 year university	1399 (8.4)	26 (8.6)		13 (8.3)	9 (8.7)		18 (8.5)	3 (6.5)		9 (7.8)	13 (9.8)		5 (11.1)	18 (8.6)	
University degree	2496 (15.0)	25 (8.3)		12 (7.7)	6 (5.8)		15 (7.0)	4 (8.7)		9 (7.0)	10 (7.5)		2 (4.4)	16 (7.5)	
**Alcohol (g/day)**															
Male	15.5	15.7	*0.868*	17.4	14.3	*0.070*	16.2	15.0	*0.648*		16.9	*0.731*	12.6	16.6	*0.331*
Female	7.7	6.2	*0.002*	6.3	5.8	*0.583*	6.0	6.7	*0.968*	6.2	5.9	*0.626*	4.6	6.2	*0.310*
**Height (cm)**															
Male	176.4	176.3	*0.400*	176.1	176.9	*0.392*	176.3	176.9	*0.773*	176.8	176.0	*0.329*	176.4	176.3	*0.817*
Female	163.6	163.3	*0.220*	162.8	163.6	*0.179*	163.4	162.6	*0.394*	162.2	164.2	*0.006*	163.1	163.3	*0.620*
**Weight (kg)**															
Male	81.7	83.8	*0.014*	83.9	82.9	*0.953*	83.1	84.5	*0.457*	84.6	82.8	*0.250*	86.2	82.8	*0.433*
Female	68.0	70.0	*0.008*	70.9	68.3	*0.112*	70.4	71.0	*0.881*	69.0	71.5	*0.154*	68.9	70.7	*0.484*
**BMI (kg/m2)**															
Male	26.2	26.9	*<0.001*	27.0	26.5	*0.481*	26.7	27.1	*0.562*	27.0	26.7	*0.591*	27.5	26.6	*0.258*
Female	25.4	26.3	*0.001*	26.7	25.5	*0.013*	26.7	27.0	*0.525*	26.2	26.5	*0.929*	25.9	26.5	*0.402*
**WHR (cm/cm)**															
Male	0.94	0.95	*0.021*	0.95	0.94	*0.027*	0.95	0.95	*0.676*	0.95	0.95	*0.482*	0.94	0.95	*0.889*
Female	0.80	0.80	*0.490*	0.80	0.80	*0.779*	0.79	0.81	*0.098*	0.80	0.79	*0.338*	0.79	0.80	*0.779*
**Waist (cm)**															
Male	94.0	96.3	*<0.001*	96.6	94.9	*0.424*	95.6	96.8	*0.575*	96.9	95.4	*0.427*	96.6	95.5	*0.586*
Female	77.9	80.1	*0.001*	80.9	78.8	*0.215*	79.9	81.9	*0.299*	79.9	80.7	*0.839*	79.0	80.6	*0.494*
**Hip (cm)**															
Male	99.4	101.2	*<0.001*	101.0	101.0	*0.602*	100.8	101.6	*0.687*	101.7	100.7	*0.391*	102.2	100.8	*0.554*
Female	97.8	100.2	*<0.001*	101.2	98.8	*0.091*	100.4	101.0	*0.872*	99.5	101.4	*0.287*	99.6	100.9	*0.380*

## Results

### Distribution of risk factors in cases and rest of cohort

As shown in Table 1, CRC cases were slightly older (p <0.001 for both men and women), of higher weight (p = 0.014 for men and p = 0.008 for women), had a higher BMI (p = <0.001 for men and p = 0.001 for women), a higher waist circumference (p < 0.001 for both men and women), and a higher hip circumference (p < 0.001 for men and p = 0.001 for women) and a higher WHR in men (p = 0.021), than the rest of cohort. Among women, cases had a higher level of education (p = 0.009), and had a lower intake of alcohol (p = 0.002) than the rest of cohort. There was a significant association between beta-catenin positive tumours and level of education (p = 0.019), a higher BMI (p 0.013) in women and with WHR (p = 0.027) among men. Cyclin D1 positive tumours were associated with higher age (p = 0.001) in women. Furthermore, p53 positive tumours were associated with height (p = 0.009), more frequent among never-smokers in women (p = 0.009), and MSS was associated with higher age in both men (p = 0.029) and in women (p = 0.024).

### Hazard ratios of CRC risk defined by different tumour characteristics in women

Associations of anthropometric factors with tumour biological parameters in women are shown in Table 2 (height, weight, hip) and Table 3 (BMI, WHR, waist). In women, a high height was associated with risk of cyclin D1 positive (p_trend_ =0.031), and p53 negative (p_trend_ =0.004) CRC. The risk of p53 negative tumours was highest in the top quartile of height (p for heterogeneity = 0.013). A high weight was associated with beta-catenin positive (p_trend_ =0.010), cyclinD1 positive (p_trend_ =0.019), p53 negative (p = 0.004) and MSS tumours (p_trend_ =0.008). Increased hip circumference was associated with beta-catenin positive (p_trend_ =0.014), p53 negative (p_trend_ =0.042) and MSS tumours (p_trend_ =0.005), but waist circumference and WHR were not associated with risk of any of the molecular subsets of CRC. A high BMI was associated with increased risk of beta-catenin positive (p_trend_ =0.004), but not beta-catenin negative tumours, with the highest risk in the top quartile (p for heterogeneity = 0.048). High BMI was also associated with risk of MSS tumours (p_trend_ =0.009).

**Table 2:** Hazard ratios of CRC risk defined by different tumour characteristics in relation to height, weight and hip circumference in women

**Tumour characteristics**	**Quartiles**	**Height**		**Weight**		**Hip**	
**Number of cases**		**Cases**	**RR**	**Cases**	**RR**	**Cases**	**RR**
Positive nuclear beta-catenin	1	31	1.00	22	1.00	19	1.00
	2	56	1.34(0.86-2.08)	39	1.50(0.89-2.52)	38	1.41(0.81-2.46)
	3	39	1.32(0.82-2.13)	46	2.04(1.22-3.41)	37	1.28(0.73-2.26)
	4	32	1.17(0.70-1.96)	51	1.87(1.12-3.10)	64	1.93(1.14-3.26)
	*p* trend		0.593		0.010		0.014
Negative nuclear beta-catenin	1	20	1.00	19	1.00	21	1.00
	2	30	1.13(0.64-2.00)	32	1.46(0.82-2.57)	21	0.72(0.39-1.32)
	3	26	1.33(0.74-2.40)	22	1.17(0.63-2.17)	30	1.03(0.58-1.81)
	4	27	1.56(0.86-2.84)	30	1.31(0.73-2.33)	31	0.88(0.50-1.55)
	*p* trend		0.113		0.599		0.985
CyclinD1 positive	1	39	1.00	35	1.00	34	1.00
	2	70	1.36(0.92-2.02)	54	1.32(0.86-2.02)	45	0.92(0.59-1.44)
	3	53	1.47(0.97-2.23)	56	1.57(1.03-2.41)	57	1.11(0.72-1.70)
	4	52	1.62(1.05-2.49)	69	1.60(1.06-2.42)	78	1.28(0.85-1.93)
	*p* trend		0.031		0.019		0.110
CyclinD1 negative	1	12	1.00	6	1.00	6	1.00
	2	14	0.80(0.37-1.73)	12	1.70(0.63-4.54)	11	1.42(0.52-3.85)
	3	12	0.89(0.39-1.99)	14	2.38(0.91-6.22)	11	1.49(0.55-4.07)
	4	8	0.61(0.24-1.53)	14	1.96(0.75-5.12)	18	2.13(0.83-5.48)
	*p* trend		0.365		0.155		0.103
p53 positive (>50%)	1	30	1.00	25	1.00	19	1.00
	2	46	1.16(0.73-1.84)	32	1.07(0.63-1.80)	32	1.19(0.67-2.10)
	3	32	1.10(0.67-1.83)	34	1.31(0.77-2.20)	34	1.19(0.67-2.10)
	4	21	0.78(0.43-1.39)	38	1.21(0.72-2.00)	44	1.31(0.76-2.28)
	*p* trend		0.431		0.370		0.364
P53 negative (<50%)	1	25	1.00	16	1.00	21	1.00
	2	40	1.39(0.82-2.37)	35	1.92(1.06-3.47)	25	0.85(0.48-1.53)
	3	33	1.64(0.94-2.84)	37	2.36(1.31-4.26)	35	1.18(0.68-2.04)
	4	39	2.17(1.25-3.76)*	45	2.36(1.33-4.21)	52	1.47(0.87-2.47)
	*p* trend		0.004		0.004		0.042
MSI	1	11	1.00	8	1.00	9	1.00
	2	14	1.01(0.46-2.23)	12	1.35(0.55-3.13)	9	0.68(2.271.71)
	3	9	0.96(0.39-2.33)	11	1.49(0.60-3.71)	13	0.9(0.40-2.21)
	4	11	1.43(0.61-3.38)	14	1.49(0.62-3.59)	14	0.81(0.34-1.90)
	*p* trend		0.477		0.387		0.864
MSS	1	41	1.00	30	1.00	27	1.00
	2	67	1.20(0.81-1.78)	58	1.64(0.05-2.55)	50	1.33(0.83-2.13)
	3	56	1.39(0.92-2.09)	55	1.79(1.14-2.80)	52	1.34(0.84-2.13)
	4	48	1.28(0.83-1.97)	69	1.87(1.21-2.88)	83	1.85(1.18-2.88)
	*p* trend		0.203		0.008		0.005

Adjusted for age, level of education, smoking habits and alcohol consumption.

*Heterogeneity analysis with p < 0.05.

**Table 3:** Hazard ratios of CRC risk defined by different tumour characteristics in relation to BMI, WHR and waist and hip circumference in women

**Tumour characteristics**	**Quartiles**	**BMI**		**WHR**		**Waist**	
**Number of cases**		**Cases**	**RR**	**Cases**	**RR**	**Cases**	**RR**
Positive nuclear beta-catenin	1	20	1.00	43	1.00	23	1.00
	2	40	1.73(1.00-2.97)	29	0.83(0.52-1.34)	33	0.97(0.57-1.67)
	3	43	1.79(1.05-3.05)	40	0.92(0.59-1.43)	51	1.40(0.85-2.30)
	4	55	2.25(1.33-3.80)*	46	1.20(0.79-1.84)	51	1.36(0.82-2.25)
	*p* trend		0.004		0.366		0.097
Negative nuclear beta-catenin	1	22	1.00	24	1.00	16	1.00
	2	29	1.20(0.69-2.09)	25	1.21(0.69-2.12)	25	1.16(0.62-2.18)
	3	28	1.06(0.60-1.87)	32	1.31(0.77-2.22)	37	1.51(0.84-2.73)
	4	24	0.89(0.49-1.61)	22	0.97(0.54-1.74)	25	1.00(0.53-1.90)
	*p* trend		0.588		0.952		0.860
CyclinD1 positive	1	35	1.00	55	1.00	31	1.00
	2	53	1.31(0.85-2.02)	48	1.07(0.72-1.58)	49	1.09(0.69-1.72)
	3	61	1.42(0.94-2.17)	62	1.12(0.77-1.62)	75	1.51(0.99-2.31)
	4	65	1.48(0.97-2.25)	49	0.98(0.66-1.45)	59	1.14(0.73-1.78)
	*p* trend		0.076		0.997		0.357
CyclinD1 negative	1	7	1.00	10	1.00	8	1.00
	2	12	1.64(0.64-4.20)	7	0.80(0.31-2.11)	7	0.69(0.25-1.90)
	3	9	1.19(0.44-3.23)	12	1.18(0.51-2.74)	12	1.08(0.44-2.66)
	4	18	2.46(1.00-6.02)	17	1.92(0.87-4.22)	19	1.81(0.78-4.21)
	*p* trend		0.071		0.066		0.063
p53 positive (>50%)	1	19	1.00	30	1.00	21	1.00
	2	33	1.51(0.85-2.67)	27	1.10(0.65-1.86)	27	0.89(0.50-1.59)
	3	38	1.63(0.93-2.84)	35	1.20(0.73-1.96)	40	1.20(0.70-2.05)
	4	39	1.65(0.94-2.89)	37	1.41(0.87-2.31)	41	1.23(0.72-2.10)
	*p* trend		0.103		0.158		0.248
P53 negative(<50%)	1	23	1.00	35	1.00	18	1.00
	2	33	1.29(0.75-2.21)	28	0.96(0.58-1.58)	29	1.16(0.64-2.09)
	3	33	1.23(0.72-2.11)	39	1.08(0.68-1.71)	49	1.77(1.03-3.06)
	4	44	1.61(0.96-2.71)	31	0.95(0.58-1.54)	37	1.28(0.72-2.27)
	*p* trend		0.091		0.953		0.236
MSI	1	10	1.00	11	1.00	6	1.00
	2	9	0.79(0.32-1.95)	10	1.08(0.46-2.55)	13	1.53(0.58-4.05)
	3	14	1.09(0.48-2.48)	14	1.22(0.56-2.70)	16	1.59(0.62-4.10)
	4	12	0.91(0.39-2.25)	10	0.96(0.41-2.27)	10	0.95(0.34-2.65)
	*p* trend		0.972		0.971		0.765
MSS	1	31	1.00	57	1.00	33	1.00
	2	56	1.60(1.03-2.50)	44	0.94(0.63-1.40)	43	0.92(0.58-1.46)
	3	55	1.51(0.97-2.36)	55	0.96(0.66-1.40)	69	1.37(0.90-2.08)
	4	70	1.90(1.23-2.93)	56	1.10(0.76-1.60)	67	1.31(0.85-2.00)
	*p* trend		0.009		0.625		0.065

Adjusted for age, level of education, smoking habits and alcohol consumption.

*Heterogeneity analysis with p < 0.05.

### Hazard ratios of CRC risk defined by different tumour characteristics in men

Associations of anthropometric factors with tumour biological parameters in men are shown in Table 4 (height, weight, hip) and Table 5 (BMI, WHR, waist).

**Table 4:** Hazard ratios of CRC risk defined by different tumour characteristics in relation to height, weight and hip circumference in men

**Tumour characteristics**	**Quartiles**	**Height**		**Weight**		**Hip**	
**Number of cases**		**Cases**	**RR**	**Cases**	**RR**	**Cases**	**RR**
Positive nuclear beta-catenin	1	40	1.00	29	1.00	26	1.00
	2	36	0.97(0.61-1.55)	42	1.41(0.86-2.29)	33	1.28(0.76-2.18)
	3	33	0.71(0.44-1.14)	35	1.15(0.69-1.93)	45	1.55(0.93-2.57)
	4	42	1.15(0.73-1.81)	45	1.53(0.94-2.49)	47	1.39(0.84-2.30)
	*p* trend		0.877		0.180		0.182
Negative nuclear beta-catenin	1	14	1.00	19	1.00	17	1.00
	2	26	2.13(1.09-4.16)	19	1.02(0.53-1.94)	11	0.65(0.30-1.40)
	3	32	1.94(1.00-3.75)	26	1.31(0.71-2.43)	33	1.87(1.02-3.42)
	4	27	1.90(0.96-3.79)	35	1.70(0.94-3.07)	38	1.46(0.79-2.70)
	*p* trend		0.122		0.048		0.036
CyclinD1 positive	1	45	1.00	40	1.00	36	1.00
	2	46	1.16(0.76-1.77)	49	1.23(0.80-1.89)	32	0.91(0.56-1.48)
	3	53	1.02(0.67-1.54)	44	1.08(0.69-1.68)	61	1.57(1.02-2.41)
	4	52	1.25(0.82-1.90)	63	1.57(1.04-2.39)	67	1.38(0.90-2.12)
	*p* trend		0.442		0.058		0.034
CyclinD1 negative	1	10	1.00	8	1.00	7	1.00
	2	15	1.48(0.66-3.34)	12	1.39(0.57-3.42)	12	1.65(0.65-4.21)
	3	13	1.00(0.43-2.32)	17	1.85(0.79-4.34)	16	2.12(0.87-5.18)
	4	17	1.52(0.68-3.43)	18	1.78(0.76-4.22)	20	1.90(0.78-4.62)
	*p* trend		0.513		0.151		0.159
p53 positive (>50%)	1	22	1.00	20	1.00	15	1.00
	2	32	1.64(0.93-2.90)	26	1.27(0.70-2.30)	22	1.48(0.76-2.90)
	3	32	1.31(0.74-2.31)	33	1.42(0.79-2.54)	38	2.29(1.23-4.26)
	4	33	1.65(0.93-2.92)	40	1.85(1.06-3.23)	44	2.12(1.14-3.92)
	*p* trend		0.191		0.026		0.009
P53 negative (<50%)	1	32	1.00	27	1.00	27	1.00
	2	30	1.02(0.62-1.70)	36	1.33(0.79-2.22)	22	0.82(0.46-1.46)
	3	33	0.84(0.51-1.39)	27	1.06(0.61-1.83)	39	1.36(0.82-2.27)
	4	36	1.13(0.68-1.87)	41	1.48(0.88-2.48)	43	1.15(0.69-1.91)
	*p* trend		0.845		0.251		0.288
MSI	1	5	1.00	4	1.00	5	1.00
	2	9	2.07(0.69-6.20)	7	1.76(0.51-6.05)	5	0.94(0.27-3.26)
	3	5	0.98(0.28-3.42)	7	1.91(0.55-6.60)	6	1.06(0.32-3.51)
	4	8	1.79(0.55-5.77)	9	2.36(0.70-7.96)	11	1.45(0.49-4.32)
	*p* trend		0.630		0.176		0.431
MSS	1	48	1.00	46	1.00	39	1.00
	2	51	1.16(0.77-1.74)	51	1.09(0.73-1.65)	39	1.03(0.65-1.61)
	3	59	1.02(0.69-1.53)	52	1.07(0.71-1.62)	64	1.54(1.02-2.33)
	4	58	1.25(0.83-1.87)	67	1.38(0.93-2.05)	74	1.40(0.93-2.11)
	*p* trend		0.411		0.126		0.038

Adjusted for age, level of education, smoking habits and alcohol consumption.

**Table 5:** Hazard ratios of CRC risk defined by different tumour characteristics in relation to BMI, WHR and waist circumference in men

**Tumour characteristics**	**Quartiles**	**BMI**		**WHR**		**Waist**	
**Number of cases**		**Cases**	**HR**	**Cases**	**HR**	**Cases**	**HR**
Positive nuclear beta-catenin	1	33	1.00	29	1.00	26	1.00
	2	31	0.90(0.54-1.48)	28	1.21(0.71-2.08)	27	0.90(0.52-1.56)
	3	37	0.97(0.60-1.59)	43	1.60(0.99-2.59)	47	1.36(0.84-2.23)
	4	50	1.52(0.96-2.41)	51	2.14(1.34-3.42)*	51	1.66(1.02-2.69)
	*p* trend		0.050		0.001		0.009
Negative nuclear beta-catenin	1	25	1.00	30	1.00	21	1.00
	2	15	0.57(0.29-1.10)	18	0.78(0.43-1.45)	11	0.43(0.20-0.91)
	3	21	0.72(0.39-1.33)	26	0.98(0.57-1.68)	33	1.21(0.69-2.12)
	4	38	1.51(0.86-2.57)	25	0.96(0.54-1.69)	34	1.27(0.72-2.23)
	*p* trend		0.074		0.993		0.076
CyclinD1 positive	1	47	1.00	45	1.00	39	1.00
	2	33	0.69(0.44-1.09)	39	1.13(0.72-1.77)	29	0.62(0.38-1.02)
	3	50	0.93(0.61-1.42)	54	1.33(0.89-2.00)	63	1.22(0.80-1.84)
	4	66	1.46(0.99-2.16)	58	1.61(1.08-1.42)	65	1.40(0.93-2.10)
	*p* trend		0.019		0.015		0.009
CyclinD1 negative	1	11	1.00	14	1.00	8	1.00
	2	12	0.95(0.41-2.19)	6	0.48(0.17-1.32)	9	1.02(0.39-2.64)
	3	10	0.78(0.33-1.86)	17	1.28(0.63-2.61)	17	1.64(0.70-3.84)
	4	22	1.75(0.83-3.71)	18	1.32(0.63-2.72)	21	2.01(0.87-4.64)
	*p* trend		0.146		0.211		0.046
p53 positive (>50%)	1	23	1.00	25	1.00	16	1.00
	2	22	0.89(0.49-1.62)	24	1.18(0.66-2.12)	21	1.05(0.54-2.04)
	3	32	1.17(0.67-2.04)	33	1.43(0.84-2.44)	41	1.85(1.02-3.33)
	4	42	1.69(0.99-2.88)	37	1.72(1.02-2.91)	41	2.05(1.14-3.68)
	*p* trend		0.023		0.033		0.003
P53 negative (<50%)	1	34	1.00	32	1.00	30	1.00
	2	23	0.65(0.38-1.13)	22	0.91(0.52-1.59)	16	0.48(0.26-0.89)
	3	28	0.73(0.43-1.24)	38	1.33(0.82-2.15)	40	1.05(0.64-1.71)
	4	46	1.44(0.90-2.30)	39	1.52(0.93-2.47)	45	1.25(0.76-2.03)
	*p* trend		0.084		0.048		0.070
MSI	1	5	1.00	6	1.00	5	1.00
	2	5	0.98(0.28-3.42)	5	1.27(0.39-4.18)	4	0.73(0.20-2.72)
	3	6	0.95(0.27-3.34)	9	1.82(0.64-5.15)	9	1.58(0.53-4.75)
	4	11	2.47(0.84-7.26)	7	1.52(0.48-4.80)	9	1.50(0.49-4.65)
	*p* trend		0.082		0.344		0.272
MSS	1	53	1.00	55	1.00	43	1.00
	2	40	0.72(0.47-1.10)	40	0.90(0.59-1.39)	34	0.67(0.42-1.06)
	3	50	0.84(0.56-1.25)	58	1.14(0.78-1.67)	67	1.16(0.78-1.73)
	4	73	1.37(0.95-1.99)	63	1.36(0.93-1.98)	72	1.39(0.94-2.05)
	*p* trend		0.053		0.071		0.012

Adjusted for age, level of education, smoking habits and alcohol consumption.

*Heterogeneity analysis with p < 0.05.

High height in men was not associated with increased risk of any of the molecular subsets of CRC, but high weight was associated with beta-catenin negative (p_trend_ =0.048) and p 53 positive (p_trend_ =0.026) CRC. A high hip circumference was associated with beta-catenin negative (p_trend_ =0.036), cyclin D1 positive (p_trend_ =0.034), p 53 positive (p_trend_ =0.009) and MSS (p_trend_ =0.038) tumours. High BMI was associated with cyclin D1 positive (p_trend_ =0.019) and p 53 positive (p_trend_ =0.023) tumours, and borderline significantly associated with beta-catenin positive CRC (p_trend_ =0.050). High WHR was associated with beta-catenin positive, but not beta-catenin negative CRC (p_trend_ =0.001), with the highest risk in the top quartile (p for heterogeneity = 0.015). A high WHR was also associated with cyclin D1 positive (p_trend_ =0.015), p 53 positive (p_trend_ =0.033) and p53 negative tumours (p_trend_ =0.048). High waist circumference was associated with beta-catenin positive (p_trend_ =0.009), cyclin D1 positive (p_trend_ =0.009), p 53 positive (p_trend_ =0.003), and MSS (p_trend_ =0.012) tumours.

## Discussion

In this large prospective cohort study, we present data on associations between anthropometric factors and risk of molecular subsets of CRC, i.e. beta-catenin overexpression, expression of cyclin D1 and p53, and MSI screening status.

Positive MSI screening status has recently been demonstrated to be an independent favourable prognostic factor in the here studied cohort [40]. In the present study, no significant associations were found between any of the anthropometric measurements and risk of MSI tumours. One previous prospective study has investigated the relationship between anthropometric factors and risk of CRC according to MSI status, demonstrating an association of high BMI with MSS tumours but not with MSI tumours [35]. These data are generally in agreement with previous case control studies [8,44]. Slattery *et al.* found that MSI tumours were more common in older people, in women and in the proximal colon, and found a positive relationship between smoking and MSI, and no association between MSI tumours and obesity [8]. In this study, we found significant associations of high weight, BMI and hip circumference with MSS tumours in women. Among men, significant associations were found between increased waist- and hip ratio and hip circumference and MSS tumours. These results are consistent with previous data from Hughes *et al.*[35], and also generally in agreement with the two previous case control studies from Slattery and Campbell [8,44].

Several anthropometric factors were significantly associated with risk of beta-catenin positive CRC in both sexes; i.e. high weight, BMI and hip circumference in women, and high WHR and waist circumference in men. Differential effects on beta-catenin overexpression, attributable to the top quartiles, were seen for BMI in women and WHR in men. No anthropometric factors were associated with beta-catenin negative tumours in women, whereas in men, high weight and hip circumference were associated with betacatenin negative CRC. Accumulating evidence support a role of WNT/beta-catenin signalling in adipogenesis, obesity and metabolic disorders [45,46], as well as in carcinogenesis [14,15]. Considering the dual roles of beta-catenin in both colorectal carcinogenesis and energy metabolism, we investigated potential links between obesity and beta-catenin alterations in CRC. One former study by Morikawa *et al*. examined the associations of beta-catenin expression and obesity with survival from CRC [47], showing an improved cancer-specific survival in obese patients with tumours displaying nuclear beta-catenin localization. In non-obese patients, there were no associations between beta-catenin status and survival. Furthermore, Morikawa et al. have recently presented data on the relationship between obesity, measured as BMI, and risk of CRC according to beta-catenin status, whereby the results demonstrate that obesity and physical inactivity are associated with a higher risk of betacatenin negative but not of betacatenin positive CRC [19]. Of note, in the MDCS, beta-catenin overexpression has been demonstrated to be significantly associated with favourable clinicopathological factors and a prolonged survival [40].

As regards cyclin D1 expression, the results from the present study demonstrate a significant association between a high height and weight and risk of cyclin D1 positive tumours in women. In men, significant associations were seen between high BMI, WHR, waist and hip circumference and cyclin D1 positive tumours. Notably, in order to avoid too small subgroup analyses, a dichotomized variable of negative vs positive cyclin D1 expression was used, since this cut off takes both nuclear fraction and intensity into account and has previously shown to have the strongest impact on survival [38]. We are not aware of any previous studies on the influence of anthropometric factors on CRC risk according to cyclin D1 expression. Although various studies have linked the *CCND1* G870A polymorphism to increased risk of CRC, the findings remain controversial [22,23]. The prognostic role of cyclin D1 has been investigated in several studies, however with inconsistent results [48-52]. In a previous study, expression of cyclin D1 was found to be associated with a significantly prolonged survival from CRC in men but not in women in the MDCS [38].

Lastly, the results from the present study demonstrate a positive relationship of all investigated anthropometric factors except height and weight, with p53 positive tumours in men, whereas in women, no associations were found between any anthropometric factors and p53 positive CRC. In contrast, high height, in particular the top quartile, weight and hip circumference were associated with p53 negative tumours in women. As a cautionary remark, the correlation between p53 gene mutations and p53 positivity by immunohistochemistry is not entirely concordant, and these analyses may therefore include some false positive and negative cases [53]. Previous studies on anthropometric factors and risk of CRC according to p53 expression are sparse, and with inconclusive results. Zhang *et al.* reported a possible association between p53 overexpression and obesity [54], and Slattery *et al.* have shown a positive relationship between western style diet and p53 mutations, but not between obesity and p53 mutations [31].

Taken together, while it is well documented that body size influences CRC risk, also with differences regarding sex, location, and tumour stage, the exact biologic mechanisms underlying the association between obesity and increased risk of CRC are not fully understood. A large number of studies have shown an increased risk of CRC in men, but not in women, and the complex interplay between hormonal factors and tumour biology underlying these sex differences remains to be further elucidated. Further, our results validate previous findings demonstrating significant associations of obesity and risk of microsatellite unstable, but not microsatellite stable, colorectal cancer in both sexes.

Certain methodological aspects need further attention. The validity of the anthropometric measurements is one aspect, as there may be a potential inter-observer variation. Recommendations for the nurses performing baseline examinations described how participants should be dressed, in which position the participants should be examined, and location for the estimation of waist- and hip measurements. We therefore consider the risk of misclassification of anthropometric measurements to be low. In contrast, most previous studies have used self- reported anthropometric measures.

It is also possible that participation in the MDCS was associated with body constitution, which may have lead to potential selection bias. In a previous paper, Manjer *et al*. compared BMI in the MDCS population in relation to the background population, and found an equal distribution of overweight/obesity [25]. Another aspect is the validity of collected data. As anthropometric data was assessed only at baseline, it is possible that some individuals have gained and some have lost weight. Such a misclassification is likely to lead to an attenuation of risks and, if anything, observed risks may be underestimated.

## Conclusions

The results from this large prospective cohort study demonstrate that obesity, measured by several anthropometric factors, is differently associated with beta-catenin alterations, expression of cyclin D1 and p53, and MSI screening status of colorectal tumours in men and women. While not allowing for any firm conclusions to be drawn, these findings further support that the influence of lifestyle factors on various pathways of colorectal carcinogenesis differs between sexes. Further study on this topic is encouraged in order to enable development of novel strategies for screening and prevention of colorectal cancer.

## Competing interests

The authors declare that they have no competing interests.

## Authors’ contributions

JB performed the statistical analyses and drafted the manuscript. SW collected clinical data and evaluated the immunohistochemical stainings, SB assisted with the statistical analyses and helped draft the manuscript. BN constructed the TMAs and carried out the IHC stainings. JM and JE assisted with the data collection and helped draft the manuscript. KJ conceived of the study, carried out the histopathological re-evaluation, evaluated the immunohistochemical stainings, and helped draft the manuscript. All authors read and approved the final manuscript.
